# Some Account of the Chiritmanos of Peru, and of the Medicines Sold by Them

**Published:** 1832-11

**Authors:** Wm. Bollaert

**Affiliations:** Corresponding Member of the Medico-Botanical Society of London.


TRANSACTIONS OF THE MEDICO-BOTANICAL SOCIETY OF LONDON.

*Published under the Direction of the President and Council.*

### CHIRITMANOS OF PERU.

### Some Account of the Chiritmanos of Peru, and of the Medicines sold by them. By Wm. Bollaert, Esq., Corresponding Member of the Medico-Botanical Society of London.

The following remarks I submit to the notice of the Medico-Botanical Society, not so much for their utility, as to shew the state of medicine, and its practitioners, in some parts of South America, and existing at the present time. During a residence in Peru, I had opportunities of collecting information relative to this class of people. The Chiritmanos are by some called the *travelling doctors* of Peru, and are Indians of Upper Peru, or (as it is now termed) Bolivia; their principal residence, when at home, is denominated the Tungas, where they collect the different herbs, seeds, roots, gums, &c. which gain them a living, vending them, and giving advice upon the different diseases met with during their journeys. Their advice and knowledge of diseases only extend to the remedies they happen to have with them. Their remedies are but few, and seem to be as well known (that is, their names and supposed virtues,) to their customers as to themselves. Some idea may be formed of the distances they travel, when it is stated that they go from Upper Peru to Buenos Ayres, a distance of more than two thousand miles, and they are to be met with likewise all over the coast of Lower Peru; and these journeys are chiefly performed on foot. At first starting, several go together, with asses laden, and at certain points they diverge, some taking one road, some another: at times one only may be met with, with a large wallet slung across the shoulder, containing the remedies, each done up in a little bag.

These Indians are of the middle size, of a dark copper colour, rather coarse features, and, what does not add to their beauty is the continual chewing of the "Coca," (leaves of the Erythroxylon peruvianum, with a strong alkaline ash,) which give to the teeth and mouth a dirty green colour, and unpleasant smell. Their dress is composed of a coarse cotton shirt, without a collar; smallclothes, made wide behind; a jacket of coarse cloth, of the wool of the llama; sometimes stockings are worn, but without feet; sandals of hide; a large brimmed hat, of the wool of the llama or vicunna; the never-failing and useful poncho; and, lastly, a little bag, for toasted Indian corn, charqui, or dried beef, a few capsicums, and a gourd for holding water: thus equipped, the Chiritmanos are ready for a journey of any distance, and through any sort of country. Their dress, once put on, very rarely comes off until worn out; and black is their favorite colour. Their language is one of the aboriginal dialects, pretty well defined, and is termed the Aymará; they understand Spanish, but some of them speak it very indifferently. They are not looked upon generally, by the better-informed folk in large towns, as having any pretensions to the knowledge of medicine, but there are purchased of them gum copal, gum thus, or incense, and some other resinous bodies for the churches; alum, sulphur, and a few such like things; but in the country, and villages retired from populous towns, much credit and belief is given to their remedies: they sell them at moderate prices, and give advice gratis. On a Chiritmano entering a town, it is soon known, and in a short time he is surrounded; when for every malady he has a cure, and for every sore a salve.

From what I have seen of them, they seem perfectly to understand their calling, and to be somewhat wiser than their customers. Their principal trade is in selling charms: these are seeds, &c. perforated, and hung round the neck; their stated virtues are many. The most useful article they might bring from Upper Peru is the Peruvian bark: this would, indeed, be of great service towards the coast, where agues of a very bad cast are very common: indeed, in many of the provinces of Peru toward the south, the bark is hardly known. Where it is known, it is given in powder, in large doses, mixed with old wine, with repeated draughts of lemonade, and certainly is a sovereign remedy.

These Chiritmanos sometimes perform the operation of bleeding: this is done with a very rude sort of lancet, made by fixing into a piece of wood a chip of glass, placing it on the vein, and giving it a nick with the forefinger and thumb; something like the instrument and method used in bleeding horses.

The following are the names, &c. of most of the articles that compose this travelling shop; and as to their utility, in my humble opinion, all that can be said is, that they do little good or harm; some of the medicines may have properties to recommend them, if administered in proper doses, and by experienced hands.

*Jaco:* bole, principally of oxide of iron.

*Salvia;* good for the *ayre:* this is a term for a cold. The substance is either taken in decoction, or the leaves moistened with saliva, and applied to the temples. It is a species of sage.

*Youruma;* bark of a tree, powdered, and taken as snuff in headach.

*Piedra Biscal:* this seems to be some inert earthy body: it is directed to be ground, and taken in warm water for the heartburn.

*Quena Quena:* seeds seemingly of a species of Annona: decoction of it used in headachs and tercianas, or agues.

*Contrayerva;* a species of Dorstenia: infusion in water given in pains of the stomach.

*Chacaire:* given in pains of the sides; powdered, and taken in warm water. This is the excrement of a bird called Coco.

*Suelda con Suelda;* ground into powder, and then fried in fat, made into plasters for broken bones. It takes its name from the Spanish word *soldar*, to mend or solder.

*Huachanca;* from a species of convolvulus, probably Jalapa, is used as a purgative. The dose is marked in the specimen.

*Corro*, or *Curru;* powdered, and mixed with fat and urine, used to rub the bones when painful. Seems to be the seeds in the seed-vessels of a species of the Helicteres, or screw-tree.

*Charna;* some little sticks, mixed at times with the above. (It accompanies the foregoing.)

*Venal;* for bad eyes: the leaf is chewed, and the eyes anointed with the saliva. The bad eyes, during the operation, must be placed looking at the sun.

*Colquemillo:* this is alum; used in itch or pimples on the skin: the parts affected first washed with urine, and then the alum, in fine powder, sprinkled over them.

*Chunchemuntana;* for heartburn.

*Ymale;* for jaundice, powdered, and taken in water. Seems to be a species of Veratrum.

*Raiz de la China*, or Chinese root; used in gonorrhœa, likewise when the menses do not flow regularly: given as a decoction.

*San Juanillo*, or St. John; an agreeable bitter, chewed for toothach.

*Ointment of St. Peter;* wax, grease, &c. The Chiritmanos say several rare herbs enter into its composition.

*Aceite de Maria*, or Mary's oil: a small quantity, used as a plaster, applied to the navel of females, during childbirth, to give easy labour.

*Cebo de Utrunco;* fat of a wild animal called the Utrunco, rubbed round the waist of women in labour: said to facilitate it.

*Parches;* patches or plasters: these are of various materials, but principally of leaves of favorite plants; sometimes the Coca, Ivy, Venal, &c. These are moistened with saliva, and applied to the temples in headachs, &c. At times some ointments are prepared from the leaves with fat and wax.

*Charms:* these are of various descriptions, such as the false nutmeg, Tairuvies, small red berries; another, a large black seed. These worn, prevent people from colds and coughs. Loadstone, if worn by either sex, ensures the love of those they are attached to; said likewise to attract lovers. Another property is attributed to this substance, that of keeping evil spirits from the wearer. There are other charms against witches, ghosts, &c.; some against poison likewise.

Clysters are recommended in cases of stoppage in the bowels, but of such dirty and useless substances that they need not be mentioned here.

These are nearly all the remedies that compose the wallet, or travelling shop, of the Chiritmano, and very few seem to be of any real utility.

